# The gastrointestinal nematode *Trichostrongylus colubriformis *down-regulates immune gene expression in migratory cells in afferent lymph

**DOI:** 10.1186/1471-2172-11-51

**Published:** 2010-10-17

**Authors:** Jacqueline S Knight, David B Baird, Wayne R Hein, Anton Pernthaner

**Affiliations:** 1 AgResearch Ltd., Hopkirk Research Institute, Grasslands Research Centre, Palmerston North 4442, New Zealand; 2 VSN (NZ) Ltd., 40 McMahon Drive, Aidanfield, Christchurch 8025, New Zealand

## Abstract

**Background:**

Gastrointestinal nematode (GIN) infections are the predominant cause of economic losses in sheep. Infections are controlled almost exclusively by the use of anthelmintics which has lead to the selection of drug resistant nematode strains. An alternative control approach would be the induction of protective immunity to these parasites. This study exploits an ovine microarray biased towards immune genes, an artificially induced immunity model and the use of pseudo-afferent lymphatic cannulation to sample immune cells draining from the intestine, to investigate possible mechanisms involved in the development of immunity.

**Results:**

During the development of immunity to, and a subsequent challenge infection with *Trichostrongylus colubriformis*, the transcript levels of 2603 genes of cells trafficking in afferent intestinal lymph were significantly modulated (P < 0.05). Of these, 188 genes were modulated more than 1.3-fold and involved in immune function. Overall, there was a clear trend for down-regulation of many genes involved in immune functions including antigen presentation, caveolar-mediated endocytosis and protein ubiquitination. The transcript levels of TNF receptor associated factor 5 (TRAF5), hemopexin (HPX), cysteine dioxygenase (CDO1), the major histocompatability complex Class II protein (HLA-DMA), interleukin-18 binding protein (IL-18BP), ephrin A1 (EFNA1) and selenoprotein S (SELS) were modulated to the greatest degree.

**Conclusions:**

This report describes gene expression profiles of afferent lymph cells in sheep developing immunity to nematode infection. Results presented show a global down-regulation of the expression of immune genes which may be reflective of the natural temporal response to nematode infections in livestock.

## Background

In sheep, infections with gastrointestinal nematodes are the most important individual cause of economic losses. At present, control of nematode infections is dependent on the repeated use of anthelmintics, but this constant use of drugs has enabled strong selection for drug-resistant nematode strains. Resistance to one or more of the major anthelmintic drug families is common in all major sheep producing countries, putting the economic survival of sheep production at risk [1,2].

Generating high levels of protective immunity would provide an alternative control option. A degree of immunity does develop after repeated natural infections acquired during grazing and this may be adequate to enable the host to reject incoming larvae and to eliminate existing infections. Effective immunity can also be induced artificially by repeated infection with unnaturally large numbers of gastrointestinal nematodes. For example, repeated experimental infection with L3 larvae, followed by drug treatment at a later stage of parasite development, is recognized as an effective inducer of protective immunity [3,4]. In general, the development of protective immunity to nematode infection is marked by a Th2-type cytokine response. In sheep and cattle IL-13 and to some extent IL-5, are regarded as having major roles in the induction of immunity to intestinal nematode infections [5-7].

Analysis of global changes in gene expression using microarray technology may aid the investigation of immune responses of sheep to gastrointestinal parasites. This technology has already become an important tool to examine complex biological processes in sheep complementing the extensive body of knowledge that exists for rodent and human disease models. Studies in sheep include gene expression profiling of tissues relevant to nematode infection in selection lines genetically resistant or susceptible to GIN [8-12], in breeds with natural resistance to GIN [13,14] and in non-selection lines [11,15].

The investigation of changes of gene expression profiles in the immune cells that migrate out of the intestinal environment would help to dissect the specific and essential role that these cells play in the development and dissemination of immunity to GIN. A pseudo-afferent lymphatic cannulation procedure was used in this study to enable access to large numbers of antigen presenting cells (APC), in particular dendritic cells (DC), as they migrate out of the intestinal environment. Recently, we described and validated procedures to cannulate pseudo-afferent lymphatic vessels in sheep and thereby gain access to large numbers of afferent lymph cells [16]. We also demonstrated differential expression in these cells of specific cytokine genes relevant to nematode immunity in selection line sheep [5]. This present study has expanded this earlier work by performing a genome wide screen for gene expression changes in afferent lymph cells. This global examination of the transcriptional activity of this key migrating cell population extends the knowledge derived from immune tissue studies [10,11,13]. Our results show that nematodes down-regulate the expression of an unexpectedly high proportion of immune genes in host cells migrating from local tissue environments and that this is likely to contribute to the slow development of natural immunity to nematodes. Better insights into these modulated genes and pathways should aid in identifying mechanisms linked to immune suppression and lead to the discovery of novel immunomodulants.

## Results

### Validation of sample pooling strategy and microarray data

**Figure 1:**
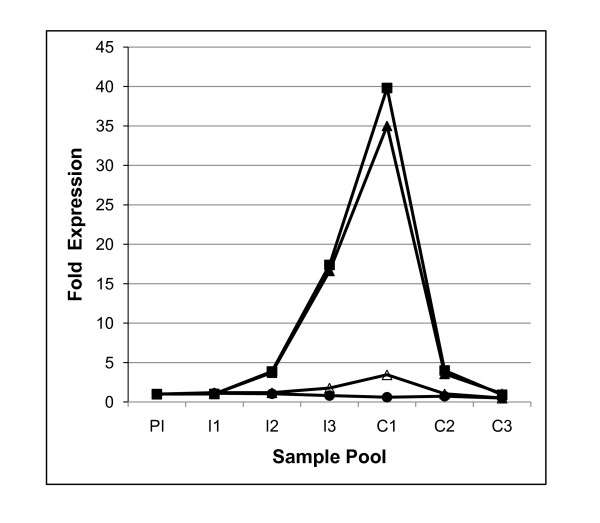
**Analysis of transcript levels of Th2 effector cytokines**. The level of IL-4 (open triangle), IL-5 (black square), IL-13 (black triangle) and IFNγ (black circle) transcripts in sample pools were measured by qRT-PCR. Expression levels were normalised to PI level. PI = week 0, I1 = week 1, I2 = week 4, I3 = week 7, C1 = week 10, C2 = week 11, C3 = week 12.

For this study, we used pseudo-afferent lymphatic cannulation procedures to continuously access immune cells migrating in lymph, draining directly from the mucosa of the sheep small intestine. The long-term collection of afferent lymph after cannulation has previously been extensively validated for sheep with GIN infections [16]. The cell population in afferent intestinal lymph contains approximately 15% DC and 85% lymphocytes. RNA from samples collected over one week, from all experimental animals in each group, was pooled. As expected, a classical Th2 cytokine profile with up-regulation of IL-5, IL-13 and to a lesser extent of IL-4, but with no changes in IFNγ transcript levels, was observed in these pooled samples with expression levels peaking after the third immunising infection and immediately after challenge (Figure 1). This is evidence that pooling RNA within treatment groups over a 7 day period did not obscure gene expression profiles shown previously to be relevant to nematode immunity.

**Figure 2:**
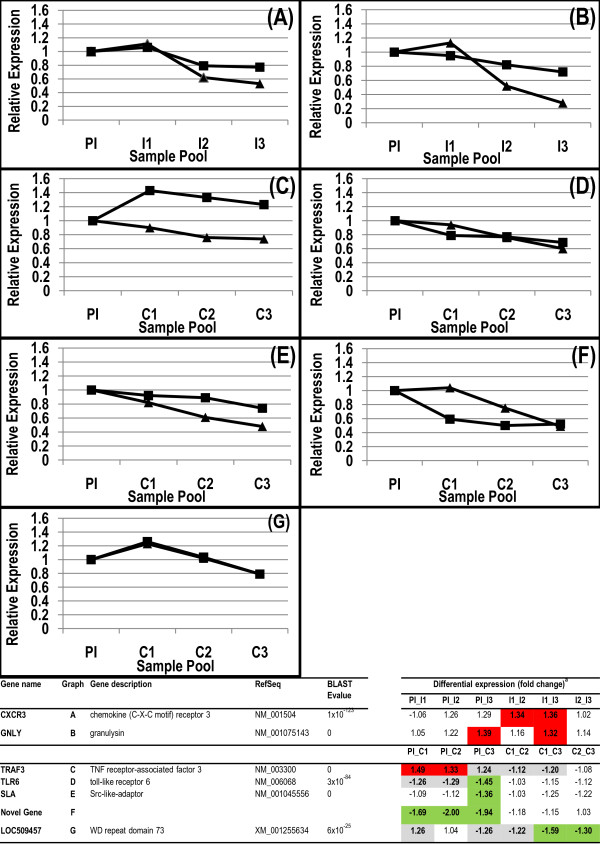
**qRT-PCR validation of microarray data**. (A) CXCR3, chemokine (C-X-C) receptor 3; (B) GNLY, granulysin; (C) TRAF3, TNF receptor-associated factor 3; (D) TLR6, toll-like receptor 6; (E) SLA, src-like-adaptor; (F) Novel Gene, no RefSeq; (G) LOC509457, predicted WD repeat domain 73. Gene expression assessed by qRT-PCR (black triangle) is expressed relative to the PI level. A time course of microarray data (black square) was calculated from comparative values and is expressed relative to the PI level. Comparative values used for this calculation are tabulated. ^a^Differential expression of genes in pooled samples PI_I1, PI_I2, PI_I3, I1_I2, I1_I3, I2_I3 or PI_C1, PI_C2, PI_C3, C1_C2, C1_C3, C2_C3 where treated as described in Methods. Down regulated genes with more than a 1.3-fold decrease (-1.3) and a P value < 0.05 are highlighted green. Up-regulated genes with > 1.3-fold increase and a P value < 0.05 are highlighted red. Genes where the change is less than a 1.3-fold decrease or increase and with a P value < 0.05 are highlighted grey. The procedure and reagents used are described in Methods.

To confirm the microarray data, the levels of 7 randomly selected genes in the sample pools were assessed by qRT-PCR. The genes assessed were CXCR3 and GNLY, identified during the repeated immunising infections, and TRAF3, TLR6, SLA, LOC509457 and a novel ovine gene identified during parasite challenge. These data showed similar changes over time between transcript levels assessed by qRT-PCR or by the microarray (Figure 2) demonstrating the validity of expression profile analysis using microarray technology. In some instances a number of different ESTs mapping to the same human or bovine gene were spotted onto the microarray. In the majority of cases the changes observed were similar for all ESTs (for example HSP90AA1, 6 ESTs; HSPA8, 6 ESTs; HLA-DQB1, 2 ESTs; YARS, 2 ESTs; ATP2B4, 2 ESTs; CD151, 2 ESTs; CD47, 2 ESTs; ACTG2, 2 ESTs; see Additional files 1, 2 and 3) further validating the array experiment.

### Analysis of modulated genes

Filters were applied, and further analysis was carried out on genes with statistically significant (P < 0.05) changes in expression levels in at least one comparison. In total, 2603 unique ESTs were modulated during the immunising infections, or the subsequent challenge. This list included 188 genes, modulated more than 1.3-fold (up or down), involved in immune function.

During the development of immunity to *T. colubriformis *the levels of 2213 ESTs with either a bovine or human homologue were statistically significantly (P < 0.05) modulated. During challenge this number was 1066 (Table 1). Of these 549 and 373 ESTs were modulated more than 1.3-fold (up or down) in at least one comparison during respectively the immunising or challenge infections. The greatest number of ESTs were modulated when Immunising infection 1 was compared to Immunising infection 2 (1353; I1_I2), and when the Pre-infection sample was compared to either Challenge infection week 2 (430; PI_C2) or to Challenge infection week 3 (498; PI_C3; Table 1). Changes in gene expression levels were modest with maximally a 1.9-fold increase and 2.6-fold decrease during the immunising infections and a 3-fold increase and 3.4-fold decrease during the challenge infection. Analysis of all ESTs modulated more than 1.3-fold showed that many more were down regulated than up regulated (Table 1).

**Table 1:** Numbers of ESTs significantly modulated during immunising and challenge infections (P < 0.05)

Immunising Infection							
	**Unique ESTs**	**Comparison^a^**					

		**PI_I1**	**PI_I2**	**PI_I3**	**I1_I2**	**I1_I3**	**I2_I3**

ESTs with RefSeq	2213	269	743	718	1353	650	193

Modulated more than 1.3-fold (up or down)	549	-	-	-	-	-	-

Down regulated (> 1.3-fold)	-	20	109	115	277	81	8

Up regulated (> 1.3-fold)	-	18	32	58	62	33	8

							

**Challenge Infection**							

	**Unique ESTs**	**Comparison^a^**					

		**PI_C1**	**PI_C2**	**PI_C3**	**C1_C2**	**C1_C3**	**C2_C3**

ESTs with RefSeq	1066	285	430	498	152	192	309

Modulated more than 1.3-fold (up or down)	373	-	-	-	-	-	-

Down regulated (> 1.3-fold)	-	55	105	122	23	23	47

Up regulated (> 1.3-fold)	-	32	42	54	14	16	32

^a ^Comparisons are described in Methods

### Modulation of gene expression during repeated truncated immunising infections

One hundred and two significantly regulated individual ovine ESTs mapping to 98 different Human or Bovine Reference genes, associated with immune function, were modulated during the immunising infections. These genes were predominantly represented in the functional groupings of inflammatory response, immune cell trafficking, antigen presentation, cellular movement, mammalian immunological disorder, cell-mediated immune response, humoral immune response and infection mechanism (Ingenuity Pathways Analysis, P < 0.02). Repeated immunising infections resulted in the down-regulation of the majority of immune genes (82 out of 102 ESTs, Additional file 1). The most consistently (significant modulation in more than one comparison) and strongly down-regulated genes were TNF receptor associated factor 5 (TRAF5, 4/6 comparisons; -2.5 fold in I1_I3), cysteine dioxygenase (CDO1, 2/6 comparisons; -2.22-fold in I1_I2), hemopexin (HPX, 3/6 comparisons; -2.01-fold in PI_I3) and the major histocompatability complex Class II DM protein (HLA-DMA, 4/6 comparisons; -1.94- fold in PI_I3). For all four genes, the maximal decrease in transcript levels occurred by the first week of the second immunising infection. The most highly up-regulated genes were the interleukin-18 binding protein at the first week of the third Immunising infection (IL18BP, 1/6 comparisons; 1.62-fold in PI_I3) and ephrin-A1 (EFNA1, 2/6 comparisons; 1.56-fold in PI_I3, Additional file 1). There was some additional down-regulation of TRAF5 expression during the challenge infection (PI_I3, -2.43 compared to PI_C3, -3.41) but for the other genes, maximum down-regulation had occurred by the third immunising infection. The up-regulated genes EFNA1 and IL18BP were not significantly modulated in the challenge infection data set.

The canonical pathways (Acute Phase Response signalling, P = 2.9 × 10^-6^; Caveolar-mediated Endocytosis, P = 1.61 × 10^-5^; Antigen Presentation, P = 8.05 × 10^-5^; Ubiquination-Proteosome System, P = 1.59 × 10^-3^; IL-4 signalling, P = 1.02 × 10^-3^) presented in Additional file 2 had the lowest significance levels, an indication of the association between the pathway and the data set. The expression of genes associated with these pathways tended to be modulated to the greatest degree between the first week of the first Immunising infection (I1) and the first week of the second Immunising infection (I2).

The decrease in gene expression in early steps of the acute phase response is consistent with the down-regulation of genes encoding TRAF6, TCF4 and STAT3, and up-regulation of HNRNPK. Modulation of transcript levels of genes encoding the acute phase response proteins FGG, HPX, C4, CRP and A2M (decrease) and RBP (increase) also support down-regulation of this response. Contrary to this, was the decrease in ALB, AMBP, APOH, ITIH2 and increase in ITIH4, FTL and LBP transcript levels.

Genes encoding proteins involved in Caveolar-mediated Endocytosis, such as the integrins (ITGA3, ITGB1, ITGA9, ITGAV), the protein kinase, FYN, the regulatory GTPase RAB5C, beta-2-microglobin (B2M), CD48 and albumin (ALB) were all down-regulated, suggesting a down-regulation of this pathway. In contrast, the levels of DMN2 encoding the GTPase dynamin 2 and ACTG2 encoding actin increased. The genes encoding major histocompatability Class II proteins (HLA-DMB, HLA-DRA, HLA-DRB1, HLA-DMA) responsible for MHC Class II antigen presentation were down-regulated as were the genes expressing TAP2, a transporter protein and PSEM2 a component of the immunoproteosome involved in presentation of antigen by major histocompatability complex Class I suggesting Antigen Presentation via both the Class I and Class II pathways was affected during repeated truncated immunising infections. Further, a number of genes encoding ubiquitin-proteosome system components responsible for targeting, via conjugation of multiple ubiquitin units, of proteins for degradation by the proteosome were modulated during the development of immunity. The expression of genes encoding the ubiquitinating enzymes, UBE2D2 and UBE4A decreased while that of UBE2J1 increased. Of the genes encoding proteins involved in degradation; PSMD5 and USP15 were decreased while USP10 and USP4 increased. Transcription of genes encoding the heat shock proteins, HSPA5, HSPA8, and HSP90AA1 was consistently reduced. The major histocompatability proteins, beta-2-microglobin (B2M) and the low affinity IgE receptor (FCER2 or CD23), all associated with IL-4 signalling, were down-regulated while the ribosomal S6 kinase (RPS6KB2) was up-regulated in this data set.

### Modulation of gene expression during challenge infection

Of the ESTs significantly regulated during the challenge infection, 121 ovine ESTs mapping to 113 different Human or Bovine Reference genes were linked to immune functions (Additional file 3), inclusive of genes associated with the inflammatory response, immune cell trafficking, inflammatory disease, antigen presentation, humoral immune response, cell-mediated immune response, lymphoid tissue structure, infection mechanism, immunological disease and apoptosis of eukaryotic cells (P values < 0.02, Ingenuity Pathways Analysis Functional groupings). The major trend during challenge was for a down-regulation in the expression of these immune-associated genes, but the degree of down-regulation was not as extensive when compared to the immunising infections (Additional files 1 and 3). The most consistently up-regulated immune gene was selenoprotein S (SELS, 3/6 comparisons). Expression increased 1.54-fold in the first week of the Challenge infection, compared to the Pre-infection, and 1.75-fold in the third week after Challenge infection, compared to the Pre-infection sample. This gene was not significantly modulated in the immunising infection data set. TNF receptor associated factor 5 (TRAF5) had the most consistently (3/6 comparisons) down regulated transcript levels. Levels decreased 2.4-fold in the first week of the Challenge infection, compared to the Pre-infection, and 3.4-fold after the third week, compared to the Pre-infection sample (Additional file 3). Over all, the most substantial fold-change for all genes modulated during the challenge infection tended to occur when the first week of the Challenge infection (C1) was compared to the Pre-infection (PI) sample pool.

The most significant canonical pathways associated with this data set were Acute Phase Response Signalling (P = 4.46 × 10^-8^), Complement Signalling (P = 4.5 × 10^-3^) and IL-4 Signalling (P = 1.22 × 10^-2^). During the challenge infection 10% of genes encoding proteins involved in the acute phase response were modulated (Additional file 4). Consistent with a decreased acute phase response were the down-regulation of STAT3 and RIPK1, up-regulation of HNRNPK and the modulation of acute phase response proteins C9, ITIH3, HPX, HP and ALB ESTs. Inconsistent with the decreased acute phase response was the observed increase in the expression of TCF4, encoding transcription factor 4, and the down-regulation of acute phase response proteins AMBP, TF, ITIH2 and AHSG. A number of the key components of the complement system (C1QC, C3, C9, C7) were down-regulated during the challenge infection. The interleukin 4 receptor (IL4R) was consistently up-regulated when either Challenge infection 1 or 2 pools were compared to the Pre-infection pool. Major histocompatability proteins (HLA-DMA, HLA-DQB1, B2M) and the low affinity IgE receptor (FCER2 or CD23), end products of IL4 signalling, were all down-regulated.

## Discussion

We deliberately interrogated a large set of ESTs derived primarily from DCs on the arrays in order to maximise our understanding of immune gene expression in a natural cell pool which is highly enriched for DC. These cells migrate out of the intestinal mucosa which forms the inter-face between host and nematode, into mesenteric lymph nodes, where immunological responses are initiated. This study describes global gene expression profiles of afferent lymph cells during the development of immunity to a gastrointestinal parasite. This understanding is crucial for the development of immune modulatory treatments, as well as for new treatment such as vaccines to control parasite infections.

For this data we employed a pooling strategy of individual samples. Analysis of cytokine gene expression in these pools by qRT-PCR revealed the expected over-expression of Th2-type cytokines. This is evidence that the more persistent modulations of gene expression are not obscured by this pooling strategy.

Due to the difficult nature of experiments involving collection of lymph for 13 weeks, not all the animals were patent for the entire term of the experiment. Therefore we cannot exclude some degree of bias at the late stages of the experiment.

It is well established that many helminths are able to produce a whole range of bioactive molecules that modulate the immune response of a host [17]. Of these molecules, glycans have been shown to be key players in inducing Th2-type and anti-inflammatory responses [18-20]. Our data not only showed a down-regulation of many individual genes involved in immunity but also are indicative of a down-regulation of entire immunological pathways, although it remains to be determined what aspects of the changes in gene expression are due to the natural response of the host to GIN infection. These data are consistent with *in vitro *studies of helminth-stimulated monocyte-derived dendritic cells (MoDC) in which gene expression profiles are also indicative of non-responsiveness [21,22]. This may be due to the production of immunomodulatory molecules by the parasite effectively inducing an anti-inflammatory environment sympathetic to parasite persistence [23]. Soluble molecules from the eggs of the helminth *Schistosoma mansoni *(SEA) by themselves have a minimal effect on MoDC but are capable of suppressing the expression of pro-inflammatory genes usually present after LPS stimulation. It is hypothesised that suppression of LPS activation may in part be due to increased production of IL-10, an anti-inflammatory cytokine [22,24]. In general, stimulation of MoDC with helminth derived products inhibit or partly inhibit DC maturation assessed by measurement of DC maturation markers such as CD86, CD40, OX40L, CD80 and MHC Class II molecules [24-28]. Our data clearly show that under *in vivo *conditions the antigen presentation pathway is also significantly down-regulated during repeated truncated immunising infections, but not during challenge of immune lambs. It remains to be determined if inhibition of DC maturation also occurs during natural infection with gastrointestinal nematode parasites, as seen in *in vitro *experiments. The predominant Th1-type response of MoDC stimulated by bacterial lipopolysaccaride (LPS), marked by increased chemokine production [29] as well as expression of genes encoding proteins related to cell structure, antigen presentation and IFN-inducible proteins [30], was not detected at any stage, in lambs repeatedly infected and then challenged with intestinal nematodes.

Previous studies in sheep analysing changes in global transcript levels associated with parasite resistance or infection have involved analysis of abomasal, intestinal and associated immune tissues. In this study transcript levels have been assessed in immune cells that migrate directly out of the tissue which harbours an intestinal nematode parasite. Microarray analysis of gene expression profiles in both abomasal tissue and lymph nodes has previously been used to investigate *Haemonchus contortus *resistance in two different sheep breeds and suggest that the more resistant breed has greater expression of genes associated with the inflammatory response, gut motility, and cell differentiation and proliferation [13]. The comparisons of gene expression in intestinal tissue and associated immune tissues from sheep lines genetically resistant or susceptible to GIN (*H. contortus*, *T. colubriformis*) [8,10,11], or by the sequential biopsy of abomasal mucosa during *H. contortus *infection [15] suggest that genes involved in acquired immunity, oxidative stress, apoptosis and mucosal function are modulated. In these studies modulation of expression of ITGB1, THBS1 and GPX1 was found to be in common with this study. The production of oxidants by the host, are thought to be anti-parasitic [31] and as such there is also a requirement for antioxidants. The levels of the antioxidant glutathione are thought to fluctuate during GIN infections [32]. A previous study [10] and this study have found that transcription of glutathione peroxidise (GPX1) increases during GIN infection, thus supporting an increase in antioxidant activities. Keane et al [9] found the most highly expressed genes in intestinal tissue of susceptible animals to be those involved in protein degradation including a ubiquitin-like protein.

Ingenuity Pathway Analysis of data presented in this study is indicative of a down-regulation of many genes involved in protein ubiquitination, caveolar-mediated endocytosis, both MHC Class I and Class II antigen presentation during immunising infections, which together could contribute to the natural slow development of protective host immunity to gastrointestinal nematode parasites. Protein ubiquitination is not only associated with the degradation of damaged proteins but in the regulation of cellular processes including immune responses such as antigen presentation and activation of pro-inflammatory responses via the transcription factor NF-κB (reviewed in [33]). Intriguingly, the heat shock proteins encoded by HSP90AA1 and HSPA5 enhance DC maturation. The former has also been shown to enhance the TLR-mediated production of pro-inflammatory cytokines [34,35]. In this study, the transcription of both the HSP90AA1 and HSPA5 genes was down-regulated during repeated immunising infections suggestive of an inhibition of DC maturation. Suppression of DC maturation is well known from *in vitro *studies in which helminth products have been shown to regulate DC cytokine and cell surface molecule maturation markers [24,25,28]. It remains to be established whether or not heat shock proteins HSP90AA1 and HSPA5 are involved.

A number of genes encoding proteins involved in acute phase responses were also modulated in this data set. Acute phase proteins (APPs) are predominantly synthesised in the liver. However, their levels can also be modulated in non-hepatic tissues such as the lymph node [36,37] and possibly in migrating lymph cells. The expression of genes encoding a group of APPs were down-regulated, consistent with routine function during the acute phase response. One of the most highly down-regulated genes during the development of immunity was hemopexin (HPX). The signalling adaptor molecule TRAF6 and the transcription factor STAT3, both involved in elicitation of an acute phase response, were down-regulated while HNHKPK, a negative regulator of the response was up-regulated. The regulation of these genes was observed during both the development of immunity and the challenge infection. Modulation of these genes is consistent with a down-regulation of the acute phase response.

Signalling is an essential process required for the integration of immune responses. A number of proteins involved in such pathways were significantly modulated during the development of immunity to parasite infection. TRAF proteins function as adaptor proteins in TNF signalling pathways and may play a role in immunity to gastrointestinal parasites [38]. TRAF5 transcript levels where the most significantly and consistently modulated during repeated immunising infections and remained low during the challenge infection. TRAF5 signalling is involved in the development of Th2-type responses, as exemplified by knockout mice studies in which TRAF5^-/- ^mice showed an enhanced Th2 phenotype [39]. Our data clearly show the development of Th2-type effector cytokine response during repeated immunising infections which is supported by the strong down-regulation of TRAF5 signalling and the up-regulation of the IL-18 binding protein. Interleukin-18 binding protein (IL-18BP) binds and inhibits the biological activity of the pro-inflammatory Th1-response promoting cytokine IL-18 [40]. IL-18 has also been implicated in the susceptibility of mice to *Trichuris muris *infection which is achieved via down-regulation of the production of IL-13 [41]. The genes IL22RA1, IL20RA, IL10RB and IL10RA, encode receptors for members of the IL-10 cytokine family (IL-10, IL-20, IL-22, IL-26). IL10RB associates with IL22RA1 or with IL20RA to form respectively the IL-22 or the IL-26 receptor while IL20RA and IL20RB form the IL-20 receptor. The transcription of IL10RB, IL22RA1 and IL20RA were down-regulated during the development of immunity to *T. colubriformis*. Both IL-22 and IL-26 have been shown to promote pro-inflammatory gene expression in intestinal epithelial cells [42,43]. Recently the removal of IL-22 was shown to have a protective effect in gut infected with the parasitic protozoan *Toxoplasma gondii *[44]. It is feasible that these cytokines could also be involved in the development of immunity to *T. colubriformis. *IL-22, IL-20, IL-26 and IL-18 have all been implicated in inflammatory conditions such as Crohn's disease and Psoriasis in humans [42,43,45,46]. IL-4, a Th2 cytokine, plays a critical role in the host's defence against gastrointestinal parasites [47]. Our data show an up-regulation of the IL-4 receptor (IL4R) during the challenge infection indicative of a Th2-type response. Other genes such as those encoding major histocompatability proteins, the low affinity receptor for IgE (FCER2 or CD23) and beta-2-microgobulin (B2M) were down-regulated. These genes are associated with, but are not exclusive to, IL-4 signalling.

Under stress conditions, endoplasmic reticular function is impaired resulting in activation of the transcription factor NF-κB and the expression of pro-inflammatory cytokines. Maintenance of endoplasmic reticular integrity under such conditions can involve selenoprotein S (SELS). A genetic variant of the SELS gene with impaired expression and suppression of transcript levels with a SELS small interfering RNA suggests that a decrease in SELS expression is linked to an increase in pro-inflammatory cytokine levels [48]. Any elevation in SELS transcript levels during immunising infections and during parasite challenge is therefore consistent with the ability of parasites to induce the suppression of an inflammatory response. Cysteine dioxygenase (CDO1) may be involved in the inflammatory response to oxidative stress via the regulation of taurine metabolism. Regulation of this involvement is however thought to be via a post-translational mechanism [49] and so further investigation would be required to determine the significance of the modulation of this gene during the development of immunity to gastrointestinal parasites.

## Conclusions

Our findings present a global picture of the changes in gene expression in cells trafficking in afferent lymph over extended periods of time during the development of immunity and challenge with the gastrointestinal parasite *T. colubriformis*. This report describes gene expression profiles in immune cells draining directly from the intestinal mucosa; the interface between the host and the nematode. Data are suggestive of a down-regulation of the expression of immune genes, a down-regulation that may be relevant to the development of immunity. These results lay the groundwork for further studies on nematode mediated immune-modulation which results in this slow development of immunity to gastrointestinal parasites. In particular, it remains to be determined if the observed changes in gene expression are also seen on a protein level resulting in functional changes of pathways.

## Methods

### Animals and experimental design

**Figure 3:**
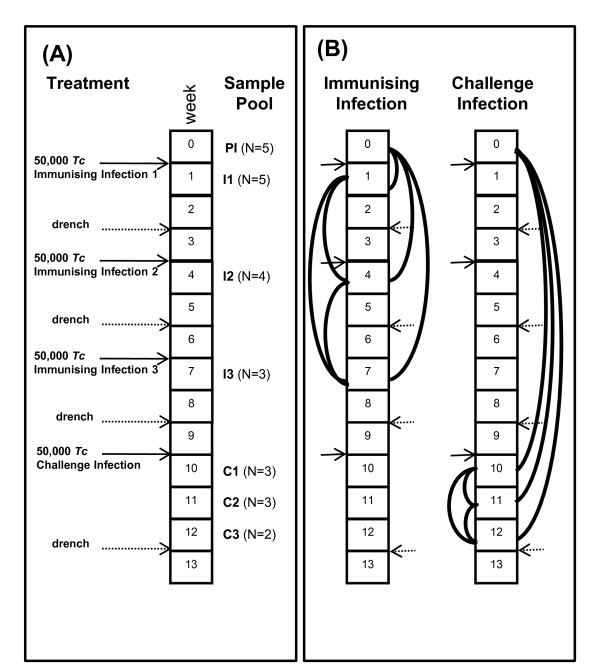
**Experimental Design**. (A) Truncated Immunising infection and Challenge protocol. Daily afferent lymph samples were collected over a 13 week period. RNA was pooled (sample pools) after extraction from afferent lymph samples from weeks labelled PI (Pre-infection, week 0), I1 (Immunising infection 1, week 1), I2 (Immunising infection 2, week 4), I3 (Immunising infection 3, week 7), C1 (Challenge infection, week 10), C2 (Challenge infection, week 11) and C3 (Challenge infection, week 12). N; number of animals in each sample pool, solid arrows show infection with 50,000 *T. colubriformis *(*Tc*) and dashed arrows show drench treatment. (B) Hybridisation of RNA pools. Solid lines show the comparisons made; solid arrows show nematode infection and dashed arrows show drench treatments.

All animal experiments and surgical procedures were approved by the Animal Ethics Committee of the Wallaceville Animal Research Centre. Animals were raised nematode free and were fed on a standardised diet consisting of sheep nuts and Lucerne chaff. Five outbred nematode naïve female Romney lambs were surgically fitted with lymphatic cannulae to enable continuous collection of afferent lymph for up to 13 weeks [16]. Lambs were immunized by orally infecting them with 50,000 *T. colubriformis *L3 larvae and the infection was terminated with oxfendazole (5 mg/kg; Systamex^® ^COOPERS) 2 weeks later. After a resting period of one week, the oral infection followed by drenching procedure (= 'truncated-immunising infection') was repeated twice. A final drench was given at week 9 and sheep were then challenged with 50,000 *T. colubriformis *a week later. The challenge infection was allowed to develop for 3 weeks. Faecal egg counts (FEC) of zero, determined at week 13, demonstrated the development of protective immunity. This regime of truncated-immunising infections and challenge infection is shown diagrammatically in Figure 3A.

### Sampling of lymph

Lymph was collected continuously into sterile flasks containing heparin (Sigma-Aldrich, St Louis, MO) and antibiotics (penicillin and streptomycin; Sigma). In addition, up to four samples of 5 to 10 ml of fresh lymph were collected under aseptic conditions over a period of 15 to 30 min daily, into sample tubes containing heparin to allow sampling of cells for RNA extraction. Cells (approximately 10^8^) were promptly separated from lymph plasma by centrifugation at 400 g for 3 min, washed once in cold PBS and re-suspended in RNAlater (Ambion, Austin, TX) to preserve integrity. Samples were stored at -20°C until subjected to RNA extraction.

### Parasitology

Infective *T. colubriformis *larvae were cultured under standard procedures from eggs obtained from faeces of a mono-specifically infected sheep. Faecal egg counts were performed on each animal at weekly intervals throughout the duration of the experiment using the modified McMaster method [50].

### Microarray preparation

Ovine cDNA libraries were generated and single pass sequenced to generate expressed sequence tags (ESTs). The amplification of cDNAs and array preparation were as described previously [8]. An ovine microarray containing 10,458 amplified cDNAs and 119 control spots was used. The majority (5215) of cDNAs printed on the array were derived from immune tissue libraries, including isolated dendritic cells (5053) with the remaining 162 from lymph node tissue, Peyers Patches and mucosal lymphoid tissue. The other cDNAs printed onto the array were from gall bladder (2638), liver (1748), foetal and reproductive tissue (734), wool follicles (96) and skin cells (30).

### RNA isolation, fluorescent labelling and slide hybridisation

Total RNA was isolated from individual lymph cell samples using Trizol (Invitrogen, Carlsbad, CA) and the appropriate RNA samples pooled and further purified using an RNeasy kit (Qiagen, Hilden, Germany). RNA concentrations were determined by the spectrophotometric measurement of absorption at 260 nm and every RNA preparation was assessed for integrity and the absence of genomic DNA contamination by agarose gel electrophoresis. First strand cDNA labelled with either Cy3 or Cy5 (Amersham Biosciences, Uppsala, Sweden) was produced from 20 μg of total pooled RNA using the SuperScript Indirect cDNA Labeling System (Invitrogen). All procedures were as described by the manufacturer. Cy3 and Cy5-labelled cDNA were pooled, concentrated to 10 μl by ethanol precipitation prior to denaturation at 95°C, combining with SlideHyb glass array hybridisation buffer #1 (60 ul, Ambion) and applied to the array. Array pre-hybridisation and hybridisation conditions, scanning and image processing were as described [8], except that hybridisation was at 52°C.

Pools (PI, Pre-infection, week 0; I1, Immunising infection 1, week 1; I2, Immunising infection 2, week 4; I3, Immunising infection 3, week 7; C1, first week after Challenge infection, week 10; C2, second week after Challenge infection, week 11; C3, third week after Challenge infection, week 12; Figure 3A) consisted of equal amounts of total RNA from each animal and for each day over a period of 7 days being pooled. Each pool was compared with every other pool (Figure 3B). In total 12 slides were hybridised to investigate the development of immunity (Immunising infections) and another 12 to investigate the challenge of immunised sheep (Challenge infection), and included slides where first strand cDNA was labelled with the opposite dye (dye swap). The number of animals contributing to each pool changed as not all animals had a patent cannula for the entire experimental period of 13 weeks (Figure 3A).

### Microarray normalisation and analysis

Data for individual slides were normalised as described previously [8,51]. ESTs with either a normalised mean log intensity < 9 and with > 6 bad (unreadable) spots out of 12 were excluded from further analysis. The standardized residual of the normalized log ratio of the mean (SR_mean) was calculated for each EST and converted to a fold change. An EST was included for further bioinformatic analysis if for one of the comparisons the change was greater than 1.3 (up or down) and the P value for this change was < 0.05. The modulation threshold of 1.3-fold was slightly more stringent than that validated and used by others [52]. A Human or Bovine Reference Sequence ftp://ftp.ncbi.nih.gov/refseq/ corresponding to each EST was determined by BLAST. Those with E values less than 1 × 10^-19 ^were included. Canonical pathways and Functional groupings were generated through the use of Ingenuity Pathways Analysis (Ingenuity Systems, http://www.ingenuity.com). All the microarray data presented in this publication have been deposited in NCBI 's Gene Expression Omnibus http://www.ncbi.nlm.nih.gov/geo/ under accession number GSE23859 for the Immunising infection data set and GSE23863 for the Challenge infection data set.

### Quantitative real-time PCR

First-strand cDNA was synthesised from the same RNA samples used in the microarray experiments. Synthesis of cDNA and the procedure used to quantitatively assess gene expression using quantitative real-time PCR (qRT-PCR) was done as described previously [5]. Oligonucleotides (Table 2) designed to amplify the ovine homologue of the src-like adaptor (SLA), chemokine (C-X-C) receptor 3 (CXCR3), TNF receptor-associated factor 3 (TRAF3), toll-like receptor 6 (TLR6), granulysin (GNLY), predicted WD repeat domain 73 (LOC509457) and a novel (no Reference Sequence) ovine EST (Novel Gene) were based on their EST sequences. Prior to qRT-PCR the identity of the EST spotted onto the array was confirmed by partial sequence analysis. Quantification of IL-5, IL-13, IL-4 and IFNγ levels within the same RNA samples were assessed as described previously [16].

**Table 2:** Oligonucleotide sequences of ovine GAPDH and a selected range of genes employed for SYBR Green real time PCR

Gene	Forward primer (5'-3')	Reverse primer (5'-3')
GAPDH	CACCATCTTCCAGGAGCGAG	CCAGCATCACCCCACTTGAT
Novel Gene	CAAGCTAAAGGCAGCATCCC	TCTCCCTCATAAGCCTGGAGC
GNLY	GGTCTGCAAAAGCAAGGCAG	TCAGAGGACCCAGGGAATCA
SLA	ACCACGGTTGGCTGTTTGAA	GCAGCTCCTCAGCCTTGTCT
CXCR3	GTGCTGACACTCCCTCTCTGG	AAAGACCCACTGGATGGCTG
TRAF3	CTTCTGTGAGACCTGCATGGG	CATTTTGGGCTGGAGGAGC
TLR6	AATGACTTTGATGCCCTGCC	CTGGGTCAAGTTGCCAAATTC
LOC509457	AAGGGATACGGGAACTTGGC	AAAGGGCTTCATTGCTGAGC

^**a **^Primers were designed to the ovine homologues of glyceraldehyde 3-phosphate dehydrogenase (GAPDH), the src-like adaptor (SLA), chemokine (C-X-C) receptor 3 (CXCR3), TNF receptor-associated factor 3 (TRAF3), toll-like receptor 6 (TLR6), granulysin (GNLY), predicted WD repeat domain 73 (LOC509457) and a novel ovine EST (Novel Gene).

## Authors' contributions

JSK performed all the RNA extractions, the microarray experiments and analysed the data using Ingenuity Pathways Analysis. DBB was responsible for microarray experiment design and performed the statistical analysis. WRH and AP conceived the study, performed the cannulation surgery, animal infections and sampling. AP co-ordinated the study. JSK and AP wrote the manuscript. All authors read and approved the final manuscript.

## Supplementary Material

Additional file 1 **Changes in immune gene expression during the Immunising infections are tabulated**. The data set is described: ^a ^Genes associated with Immune Function in the Ingenuity Pathways Knowledge Base where at least one comparison has a P value < 0.05 (shown in bold type). Differential expression of genes in pooled samples PI_I1, PI_I2, PI_I3, I1_I2, I1_I3, I2_I3 where treated as described in Methods. Down-regulated genes with more than a 1.3-fold decrease (-1.3) and a P value < 0.05 are highlighted green. Up-regulated genes with > 1.3-fold increase and a P value < 0.05 are highlighted red. Genes where the change is less than a 1.3-fold decrease or increase and with a P value < 0.05 are highlighted grey. Click here for file

Additional file 2 **The modulation of canonical pathways during Immunising infections is tabulated**. The data set is described: ^a ^Significance level. ^b ^Proportion of pathway associated genes. ^c ^Genes associated with Canonical Pathways in the Ingenuity Pathways Knowledge Base where at least one comparison has a P value < 0.05 (shown in bold type). Differential expression of genes in pooled samples PI_I1, PI_I2, PI_I3, I1_I2, I1_I3, I2_I3 are shown. Down-regulated genes with more than a 1.3-fold decrease (-1.3) and a P value < 0.05 are highlighted green. Up-regulated genes with > 1.3-fold increase and a P value < 0.05 are highlighted red. Genes where the change is less than a 1.3-fold decrease or increase and with a P value < 0.05 are highlighted grey. Click here for file

Additional file 3 **Changes in immune gene expression during the Challenge infection are tabulated**. The data set is described: ^a ^Genes associated with Immune Function in the Ingenuity Pathways Knowledge Base where at least one comparison has a P value < 0.05 (shown in bold type). Differential expression of genes in pooled samples PI_C1, PI_C2, PI_C3, C1_C2, C1_C3, C2_C3 are shown. Down-regulated genes with more than a 1.3-fold decrease (-1.3) and a P value < 0.05 are highlighted green. Up-regulated genes with > 1.3-fold increase and a P value < 0.05 are highlighted red. Genes where the change is less than a 1.3-fold decrease or increase and with a P value < 0.05 are highlighted grey. Click here for file

Additional file 4 **The modulation of canonical pathways during Challenge is tabulated**. The data set is described: ^a ^Significance level. ^b ^Proportion of pathway associated genes. ^c ^Genes associated with Canonical Pathways in the Ingenuity Pathways Knowledge Base where at least one comparison has a P value < 0.05 (shown in bold type). Differential expression of genes in pooled samples PI_I1, PI_I2, PI_I3, I1_I2, I1_I3, I2_I3 are shown. Down-regulated genes with more than a 1.3-fold decrease (-1.3) and a P value < 0.05 are highlighted green. Up-regulated genes with > 1.3-fold increase and a P value < 0.05 are highlighted red. Genes where the change is less than a 1.3-fold decrease or increase and with a P value < 0.05 are highlighted grey. Click here for file
